# Asymptomatic malaria infections and *Pfmdr1* mutations in an endemic area of Nigeria

**DOI:** 10.1186/s12936-019-2833-8

**Published:** 2019-06-27

**Authors:** Titilope M. Dokunmu, Cynthia U. Adjekukor, Omolara F. Yakubu, Adetutu O. Bello, Jarat O. Adekoya, Olugbenga Akinola, Emmanuel O. Amoo, Abiodun H. Adebayo

**Affiliations:** 1 Department of Biochemistry, Covenant University, Ota, 23401 Nigeria; 2 Department of Biological Sciences, Covenant University, Ota, 23401 Nigeria; 3 Department of Pharmacology and Therapeutics, University of Ilorin, Ilorin, 24003 Nigeria; 4 Demography and Social Statistics Unit, Department of Economics and Development Studies, Covenant University, Ota, 23401 Nigeria

**Keywords:** Asymptomatic malaria, *Pfmdr1*, Drug resistance, *Plasmodium falciparum*, Nigeria

## Abstract

**Background:**

Malaria eradication globally is yet to be achieved and transmission is sustained in many endemic countries. *Plasmodium falciparum* continues to develop resistance to currently available anti-malarial drugs, posing great problems for malaria elimination. This study evaluates the frequencies of asymptomatic infection and multidrug resistance-1 (*mdr-1*) gene mutations in parasite isolates, which form the basis for understanding persistently high incidence in South West, Nigeria.

**Methods:**

A total of 535 individuals aged from 6 months were screened during the epidemiological survey evaluating asymptomatic transmission. Parasite prevalence was determined by histidine-rich protein II rapid detection kit (RDT) in healthy individuals. *Plasmodium falciparum mdr-1* gene mutations were detected by polymerase chain reaction (PCR) followed by restriction enzyme digest and electrophoresis to determine polymorphism in parasite isolates. Sequencing was done to confirm polymorphism. Proportions were compared using Chi-square test at p value < 0.05.

**Results:**

Malaria parasites were detected by RDT in 204 (38.1%) individuals. Asymptomatic infection was detected in 117 (57.3%) and symptomatic malaria confirmed in 87 individuals (42.6%). Overall, individuals with detectable malaria by RDT was significantly higher in individuals with symptoms, 87 of 197 (44.2%), than asymptomatic persons; 117 of 338 (34.6%), p = 0.02. In a sub-set of 75 isolates, 18(24%) and 14 (18.6%) individuals had *Pfmdr1* 86Y and 1246Y mutations.

**Conclusions:**

There is still high malaria transmission rate in Nigeria with higher incidence of asymptomatic infections. These parasites harbour mutations on *Pfmdr1* which contribute to artemisinin partner drug resistance; surveillance strategies to reduce the spread of drug resistance in endemic areas are needed to eliminate the reservoir of malaria parasites that can mitigate the eradication of malaria in Nigeria.

## Background

Malaria prevalence has declined to about 216 million cases estimated in 2016 but progress in malaria reduction appears to be stagnant as reported in the World Malaria Report 2018 with the highest transmission rates still recorded in the African region [1]. Increasing drug resistance in *Plasmodium falciparum* in Southeast Asia and Africa now mitigates the use of some available artemisinin combination drugs for effective malaria eradication [1, 2]. In Nigeria, malaria transmission remains high, in South West area of Nigeria, malaria is highly endemic and transmission occurs all year round, however prevalence of malaria is often underestimated because sub-microscopic malaria is common [3]. Chemoprevention strategies for eradicating malaria, such as mass drug administration with primaquine to reduce the high reservoir of malaria infection, cannot be implemented in most malaria-endemic countries due to drug resistance and potential prevalence of glucose-6-phosphate deficiency (G6PD) [3–8]. Several studies from malaria endemic countries have reported single nucleotide polymorphisms (SNPs), including K76T on *P. falciparum* chloroquine resistance transporter gene (*Pfcrt*); N86Y, S1034C, Y184F, N1042D, and D1246Y SNPs on multidrug resistance gene 1 (*Pfmdr1*) and other loci, which confers resistance to artemisinin and its partner drugs, such as chloroquine, quinine, amodiaquine, lumefantrine, and piperaquine [9–21].

Monitoring the prevalence of these molecular markers of resistance plays a significant role in assessing effective malaria chemotherapy for reducing malaria intensities. To curb the spread of drug resistance and for effective malaria control, it is necessary to study the genetic backgrounds of parasite isolates that circulate in asymptomatic infections, which are often exposed to sub-therapeutic doses of anti-malarial drugs, as these polymorphisms alter parasite susceptibility to artemisinin combination treatment (ACT) [1]. This study aims to determine frequencies of *Pfmdr1* mutations in parasite isolates and intensities of asymptomatic malaria transmission in an endemic area of Nigeria.

## Methods

The study was carried out in Ota, South West Nigeria to evaluate asymptomatic malaria incidence and molecular markers of anti-malarial drug resistance in *P. falciparum.* Persons aged > 6 months, without any symptoms suggestive of malaria at the time malaria test was done and in persons reporting a history of fever within the previous 2 weeks participated in the community health screening for malaria prevalence and were included in the study which took place at intervals from April to May and November to December (both in dry and wet seasons) around communities in Ota, Ogun State in 2018. The participants gave informed consent and the study was approved by Covenant Health Research Ethics Committee (approval number: CHREC/010/2018). Parasite prevalence was determined by histidine-rich protein II-based rapid test kit. The minimum sample size required for the study was calculated as 384, using an estimated population of 527,242 persons living in Ota and a 5% margin of error. Molecular analysis was done at Molecular Biology Laboratory of Covenant University. Blood from positive samples were used for PCR analysis. Genomic DNA was extracted using Aidlab Blood & Tissue DNA mini kit according to the manufacturer’s instruction and primers flanking codon 86 and 1246 of *Pfmdr1* gene was used [22]. PCR amplification was done on C1000 Touch™ Thermal Cycler (Bio-Rad Laboratories Inc., USA) using PCR super-mix (New England Biolabs, USA). Table 1 shows the details of primers and PCR conditions used. Nested PCR products were restricted by enzymatic digest with specific restriction enzymes: *AflIII* (New England Biolabs, USA) was used to detect mutation causing base change from asparagine (N) to tyrosine (Y) at codon 86 while *EcoRV* (Beijing TransGen Biotech, China) was used to detect mutation causing base change from aspartic acid (D) to tyrosine (N) at codon 1246. Enzyme was incubated at 37 °C for 1 h and inactivated at 65 °C for 20 min, 10 µl of the digested product was resolved on 2% agarose gel and sized against molecular weight marker. To confirm mutation, 15  µl of amplicons were sent for sequencing (Inqaba Biotec West Africa Ltd, South Africa). Sequence alignment with reference 3D7 strain (PF3D7_0523000) was carried out using Geneious software® version 11.6. The data was analysed using SPSS software version 16 (SPSS Inc., Chicago, USA). Proportions were compared using Chi-square test at significant level of p < 0.05.

**Table 1:** Primer sequence of *Pf*mdr1 gene flanking at codon 86 and 1246

Primer	Sequence (5′ → 3′)	Direction	PCR product (bp)	Cycling condition
*Pf*mdr1 86 and 184	AGAGAAAAAAGATGGTAACCTCAG	Forward	590	95 °C for 3 min, 34 cycles of 95 °C for 45 s, 60.2 °C for 45 s, and 72 °C for 5 min
	ACCACAAACATAAATTAACGG	Reverse		
*Pf*mdr1 1246	GTGGAAAATCAACTTTTATGA	Forward	499	94 °C for 5 min, 35 cycles of 94 °C for 1 min, 53 °C for 1 min, 72 °C for 1 min
	TTAGGTTCTCTTAATAATGCT	Reverse		
	GACTTGAAAAATGATCACATT	Forward nested	409	94 °C for 5 min, 35 cycles of 94 °C for 1 min, 50 °C for 1 min, 72 °C for 1 min
	GTCCACCTGATAAGCTTTT	Reverse nested		

## Results

### Transmission intensities

**Fig. 1:**
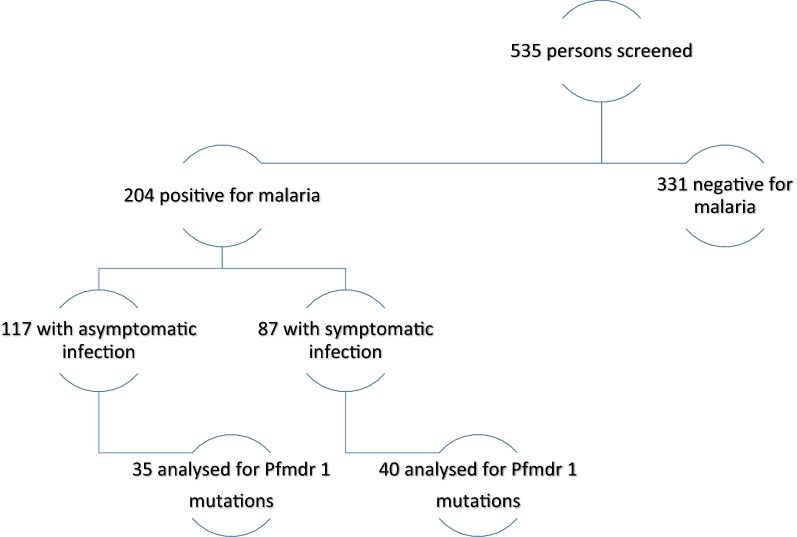
Flow chart for the study

During the study period, 535 blood samples were examined for the presence of *P. falciparum* infections by RDT. Malaria was detected in 204 (38.1%) blood samples; 87 (42.6%) infections in symptomatic carriers while 117 (57.3%) persons were asymptomatic. Overall the proportion of individuals with detectable malaria by RDT (positivity rate) was significantly higher in those with symptoms (87 of 197, 44.2%) than asymptomatic persons (117 of 338, 34.6%), χ^2^ = 4.80, p = 0.02. Based on age, overall malaria incidence was 7 of 49 (14.2%), 81 of 143 (56.6%), 60 of 195 (30.7%) and 56 of 148 (37.85) in age groups < 2, > 2–10, > 10–18, and > 18 years, respectively. Similarly, asymptomatic infection was detected in 0 of 49 (0%), 9 of 143 (6%), 54 of 195 (27.6%), and 54 of 148 (36.4%) in age groups < 2, > 2–10, > 10–18, and > 18 years, respectively. Malaria incidence was highest in children aged > 2–10 years, however asymptomatic infection was higher in older children and adults (p < 0.0001). There was no difference in prevalence between different genders. Of the 204 positive samples, 75 randomly selected samples were evaluated for the presence of *Pfmdr1* mutations. Figure 1 shows the flow chart for the study.

### *Pfmdr1* mutations

**Fig. 2:**
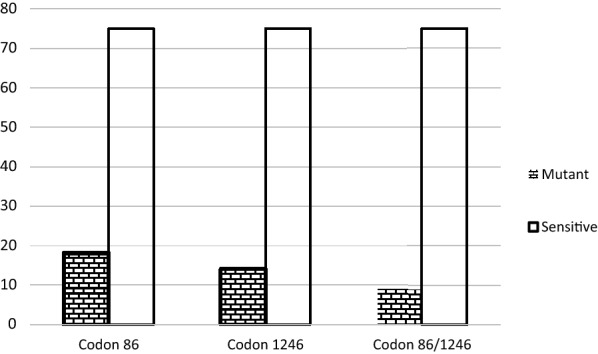
Prevalence of mutant alleles detected at codon 86 (24%) and 1246 (19%) of *Pf*mdr 1 gene in parasite isolates during the study

**Fig. 3:**
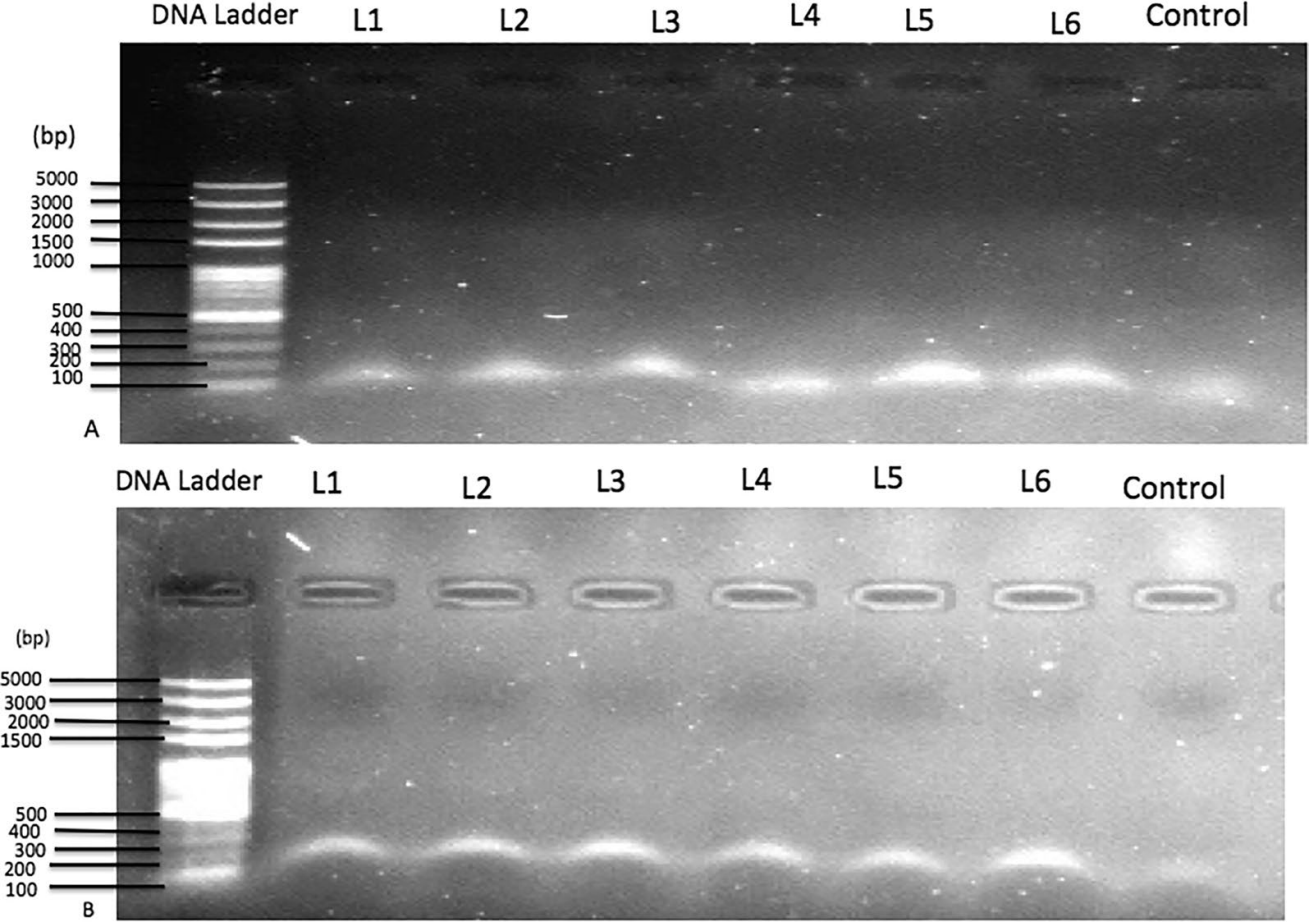
Restriction digest product for *Pf*mdr1 SNP detection at codon 86 (**a**) and 1246 (**b**)

**Fig. 4:**
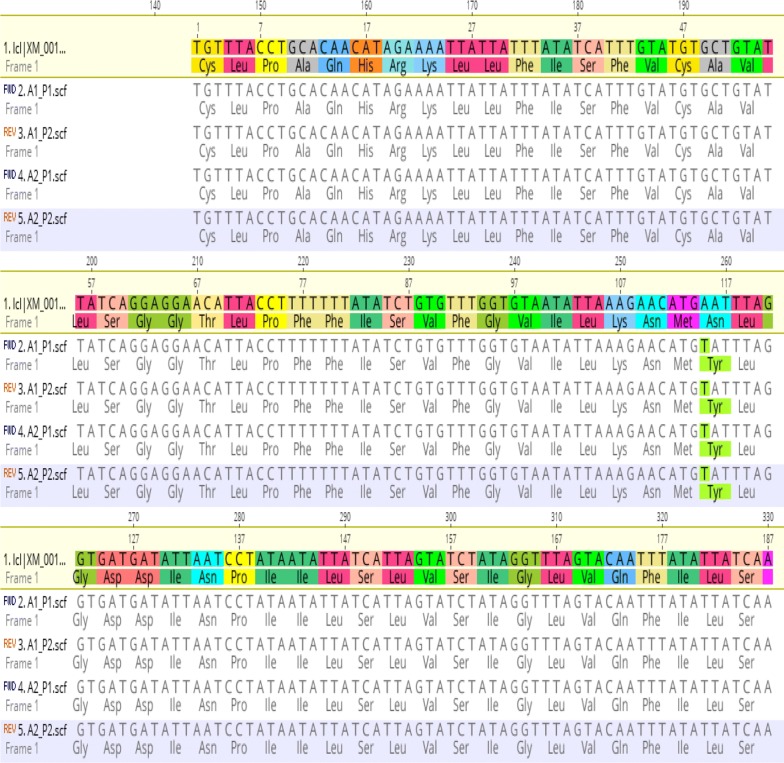
Screenshot of alignment of *Pf*mdr1 gene flanking codon 86 in 2 isolates (with mutant allele- tyrosine) compared to reference 3D7 strain (sensitive allele-asparagine)

Seventy-five positive samples selected from asymptomatic and symptomatic infections were evaluated for *Pfmdr1* mutations at codons 86 and 1246. In 18 of 75 (24%) isolates, 86Y alleles were detected. Similarly, 14 of 75 (18.6%) isolates had 1246Y mutant alleles, Fig. 2 (χ^2^ = 0.63, p = 0.42). Of the total, 9 isolates (12%) had both 86Y and 1246Y mutant alleles (Fig. 3). The proportions in asymptomatic and symptomatic infections were similar. To confirm restriction fragment length polymorphism (RFLP) digest, sequencing of few isolates with mutant 86Y allele aligned with 3D7 reference strain further confirms amino acid substitution from asparagine (N) to tyrosine (Y) (Fig. 4).

## Discussion

Malaria has persisted for several decades due to development of resistance to available anti-malarial drugs, including artemisinin derivatives and its partner drugs, and unbroken transmission in highly endemic countries [1, 2]. To achieve malaria elimination especially in endemic areas, reducing the pool of infection by asymptomatic carriers, scaling up of control measures such as chemoprevention, vector control and monitoring of resistance for effective treatment are all essential to break transmission [23, 24], which has been achieved in some countries [1, 2]. World Health Organization reported stalled progress in malaria reduction from 2015 to 2017 especially from the African region. Nigeria alone contributes 25%, the highest proportion borne by any single country to malaria burden globally, while other African countries, such as Democratic Republic of Congo (11%) and Mozambique (5%) follow [2].

Asymptomatic infection is common in endemic areas [3–7], and wide epidemiological surveys utilizing PCR are very valuable to estimate the true burden in endemic areas for planning effective malaria control strategies. Parasites isolated in untreated infections can harbour resistant genes as a tool for survival; this study reports the presence of *Pfmdr1* 86Y (confirmed by sequencing) and 1246Y mutant alleles being transmitted in asymptomatic as well as symptomatic infections. This finding is similar to reports from endemic and non-endemic areas where *Pfmdr1* has served as a molecular marker of detecting anti-malarial drug resistance [13, 14, 16, 21]. Most malaria-endemic countries are in the pre-elimination phase because local transmission is still high [2]. This is largely because malaria parasites may be harboured by persons who show no symptoms; consequently, long term carriage of asymptomatic infections promotes development of committed gametocytes, and these are picked up by mosquito, increasing malaria transmission.

This cycle of asymptomatic transmission is unhindered in a country such as Nigeria because of the lack of prophylactic chemoprevention which is not encouraged due to drug resistance in the area [25]. The current study and previously reported studies clearly demonstrate that asymptomatic malaria infection is very common [3–7] in Nigeria and other endemic countries. However, clearance of these parasite reservoirs by mass drug administration using a slowly eliminated drug and tissue schizonticide such as primaquine cannot be explored because of the limitations of drug-induced haemolysis in some regions. Reflecting on prevalence of *Plasmodium falciparum* rates over time in Nigeria to date, it can be surmised that transmission has not significantly declined if epidemiological screenings are regularly done; a rate of 57% was detected in this study which is not similar to other recent studies [26] or previous studies in Nigeria [27–29].

With this trend in other parts of Africa [30], the agenda to eliminate malaria, which aims to completely remove and eradicate the disease locally and globally, can be achieved but through elimination of circulating parasite reservoir (infection from asymptomatic persons to mosquito and to new host), a crucial step for moving endemic countries out of the pre-elimination phase. Factors that favour intense transmission, including parasite reservoir, competent vectors and suitable breeding sites and climatic conditions, are predominant in Africa; a multifaceted approach to curtail transmission through all these influential factors should be promoted.

In asymptomatic infections, a similar proportion of 86Y and 1246Y mutant alleles was present as in symptomatic infections. Asymptomatic parasite tolerance is determined by the host immunity, hence the higher proportions in adults older than 18 years. Higher prevalence of these mutations conferring resistance to chloroquine, artemether, mefloquine was previously reported in Nigeria [11, 12, 15, 16], but the findings, similar to a recent study from northern Nigeria [26], indicate a decline in the prevalence of mutations. Therefore, determining the prevalence of these molecular markers can be used to assess the degree of transmission and the impact of prophylactic chemotherapy for malaria control in Nigeria.

Mutations on the *Pfmdr1* gene play a pivotal role with variable parasite response to artemisinin, ACT and non-ACT, such as chloroquine, lumefantrine, primaquine, tafenoquine, piperaquine, and mefloquine [31–38], due to conformational changes in the transporter protein causing decrease in intracellular drug accumulation and effect on the malaria parasite.The haplotype N86Y-Y184F-D1246Y of *Pfmdr1* was reported to be susceptible to artemisinin-based combination, whereas triple mutations S1034C, N1042D and D1246Y or increased gene copy number are associated with parasite resistance to mefloquine, halofantrine and artemisinin [36]. Although this study did not assess *Pfcrt* mutation, there is known synergy between mutations on *Pfmdr1* and *Pfcrt* genes [39]. Studies in Nigeria and other areas have reported reduced in vitro and in vivo response in chloroquine-resistant isolates bearing the 76T mutation correlating strongly with *Pfmdr1* 86Y mutation [12, 14, 16, 21, 22, 38].

Although molecular markers alone may not connote resistance except resulting in drug failure, it however can predict the potential of parasites to develop drug resistance [9, 11, 12]. By implication, frequency of *Pfmdr1* mutations observed in this area is low, but malaria incidence has not decreased significantly. Including asymptomatic malaria control using combinations not previously used [40], e.g., pyronaridine-artemisinin for chemoprophylaxis in persons with malaria infections, should be considered for Nigeria to clear out circulating asymptomatic infections for complete elimination of parasite reservoir. Evidence from the study shows one in six parasite isolates bear mutant 86Y and 1246Y alleles; eliminating these with effective drugs, together with high asymptomatic parasitaemia will greatly impact malaria eradication in this endemic area.

Limitations of this study were that microscopy, the gold standard, was not used for estimating malaria prevalence and follow-up was not done to determine if asymptomatic individuals eventually became symptomatic. Secondly, histidine-rich protein II RDTs may have detected malaria antigens in recently cleared infection, and RDTs may not detect infections at very low densities in some individuals [41, 42]. Future studies evaluating asymptomatic malaria prevalence with PCR methods are recommended.

## Conclusion

There is a high malaria transmission rate in Nigeria with higher incidence of asymptomatic infections. These parasites harbour mutations on *Pfmdr1*, which contribute to artemisinin partner drug resistance; surveillance strategies to reduce the spread of drug resistance in endemic areas are needed to eliminate the reservoir of malaria parasites that can mitigate the eradication of malaria in Nigeria.

## Data Availability

All data analysed for this study is included in the manuscript, there are no additional supporting materials.

## Abbreviations

ACT: artemisinin-based combination therapy
DNA: deoxyribonucleic acid
G6PD: glucose-6-phosphate dehydrogenase
PCR: polymerase chain reaction
RDT: Rapid detection kit
RFLP: restriction fragment length polymorphism
SNP: single nucleotide polymorphism
